# The impact of PEPFAR transition on HIV service delivery at health facilities in Uganda

**DOI:** 10.1371/journal.pone.0223426

**Published:** 2019-10-09

**Authors:** Jess Alan Wilhelm, Mary Qiu, Ligia Paina, Elizabeth Colantuoni, Moses Mukuru, Freddie Ssengooba, Sara Bennett

**Affiliations:** 1 Johns Hopkins University, Bloomberg School of Public Health, Department of International Health, Baltimore, Maryland, United States of America; 2 Johns Hopkins University, Bloomberg School of Public Health, Department of Biostatistics, Baltimore, Maryland, United States of America; 3 Makerere University, School of Public Health, Department of Health Policy, Planning & Management, Kampala, Uganda; Boston University School of Public Health, UNITED STATES

## Abstract

**Background:**

Since 2004, the President’s Emergency Plan for AIDS Relief (PEPFAR) has played a large role in Uganda’s HIV/AIDS response. To better target resources to high burden regions and facilities, PEPFAR planned to withdraw from 29% of previously-supported health facilities in Uganda between 2015 and 2017.

**Methods:**

We conducted a cross-sectional survey of 226 PEPFAR-supported health facilities in Uganda in mid-2017. The survey gathered information on availability, perceived quality, and access to HIV services before and after transition. We compare responses for facilities transitioned to those maintained on PEPFAR, accounting for survey design. We also extracted data from DHIS2 for the period October 2013–December 2017 on the number of HIV tests and counseling (HTC), number of patients on antiretroviral therapy (Current on ART), and retention on first-line ART (Retention) at 12 months. Using mixed effect models, we compare trends in service volume around the transition period.

**Results:**

There were 206 facilities that reported transition and 20 that reported maintenance on PEPFAR. Some facilities reporting transition may have been in a gap between implementing partners. The median transition date was September 2016, nine months prior to the survey. Transition facilities were more likely to discontinue HIV outreach following transition (51.6% vs. 1.4%, p<0.001) and to report declines in HIV care access (43.5% vs. 3.1%, p<0.001) and quality (35.6% vs. 0%, p<0.001). However, transition facilities did not differ in their trends in HIV service volume relative to maintenance facilities.

**Conclusions:**

Transition from PEPFAR resulted in facilities reporting worsening patient access and service quality for HIV care, but there is insufficient evidence to suggest negative impacts on volume of HIV services. Facility respondents’ perceptions about access and quality may be overly pessimistic, or they may signal forthcoming impacts. Unrelated to transition, declining retention on ART in Uganda is a cause for concern.

## Introduction

The President’s Emergency Plan for AIDS Relief (PEPFAR) has played a large role in Uganda’s HIV response since 2004. In Uganda, 70% of HIV/AIDS expenditure came from international donors in 2015/16 [1], with PEPFAR being the primary source. However, in 2015, PEPFAR launched the “Geographic Prioritization (GP)” to target support to regions within countries that contribute to 80% of the national HIV burden in order to achieve the 90-90-90 targets by 2018 [2].

In Uganda, this process resulted in 734 facilities being designated to lose site-level support (“transition facilities”), another 419 being designated to receive near constant PEPFAR support (“maintenance facilities”), and 1,384 being designated for scaled-up support [3]. Site-level support varies but typically includes supervision, training, health worker salaries, and incentives, and support for outreach. Above-site support, including laboratory networks and commodity supply chains, is intended to remain in place for transitioned facilities [4]. Facilities were selected for transition on the basis of being either “low-volume”, according to PEPFAR’s data, or being located in one of ten districts in which all facilities were to be transitioned (“central support districts”). Transition officially took place over a period from October 2015 to March 2017. Whether and how health facilities are affected by the loss of PEPFAR support raises important questions about the sustainability of HIV service delivery in Uganda.

PEPFAR’s positive impact on HIV service delivery in low- and middle-income countries (LMICs), including Uganda, is widely acknowledged [5, 6]. However, as donor funding for HIV continues to decline [7], the withdrawal of donor support (commonly referred to as “transition”) and increased co-financing are increasingly likely in HIV programs. Current donor transition policies portend many transitions in the next decade [8]. However, relatively few studies have evaluated the effects of transitioning programs on HIV services [9–19]. Prior HIV transition experiences have had mixed effects. For example, the evaluation of the transition of the Gates Foundation-funded Avahan program in India identified some positive experiences in continuation of HIV service delivery and continued support for programs directed to key populations (KPs) following transition [19]. However, other studies have identified negative experiences for KP programming when donors turn programs to socially conservative governments [18, 20] and interruptions of HIV care during patient transfers to from donor-funded specialty clinics to public primary health clinics [14, 21–22].

The model of transition used by PEPFAR in Uganda differs from other cases. First, transition was limited to about 30% of supported facilities in Uganda. Secondly, PEPFAR did not withdraw support to KP programming. Lastly, PEPFAR support for national commodity, lab, and data systems remains in place, even for facilities losing site-level support. Unlike the nationwide withdrawal of support that has been common to many past transitions, PEPFAR’s transition in Uganda offers the ability to quantitatively analyze impacts at the facility level. In this study, we analyze how transition from site-level PEPFAR support in Uganda affected the availability, accessibility and quality of key HIV services at transitioned health facilities. In terms of availability, our analysis focuses on whether or not specific HIV services are still offered by transitioning facilities, or whether they have been discontinued. In terms of service accessibility and quality, we assess facility in-charges’ perceptions of changes in these dimensions of care. Finally, we assess changes in service utilization as an indicator that captures not only availability of services, but also other related factors such as quality, accessibility and acceptability [23].

## Methods

The current study emerged from a mixed methods evaluation of the PEPFAR Geographic Prioritization in Uganda and Kenya funded by United States Agency for International Development (USAID) through a Population Council Project SOAR (Supporting Operational Aids Research) grant. The parent study included document reviews, key informant interviews, longitudinal case studies at select facilities, and the survey and DHIS2 data analysis used here.

### Facility survey

A joint Johns Hopkins/Makerere University study team conducted a survey of PEPFAR-supported health facilities, offering various levels of care, in Uganda in July and August of 2017. The survey was conducted four months after the official end of the GP in March 2017 and was piloted in advance of full data collection. The survey sample frame was drawn from a list of health facilities identified by the USAID mission in Uganda as PEPFAR supported in 2014. For logistical reasons, the study team limited the sampling area for this survey to 40 districts in Northern and Eastern Uganda as well as Kampala and Wakiso districts in Central Uganda. This area contained 9 of the 10 “central support districts” as well as most facilities designated for maintenance. Kampala and Wakiso are urban districts that contain many private for-profit (PFP) sites designated for transition from PEPFAR support. At the request of the funder, we also restricted the sample frame to facilities that were supported by Implementing Partners (IPs) contracted to USAID.

We selected 28 districts from the sampling frame using a stratified cluster sampling design with three strata: 1.) 100% selection of all districts containing large transitioning facilities (i.e. health centre IVs and/or hospitals), as well as Kampala & Wakiso districts, 2.) Random sampling of remaining 11 out of 18 districts that were designated as central support or maintenance, 3.) Random sampling of 6 out of 14 remaining districts, which also contain some “low-volume” transition facilities. Within selected districts, all facilities intended for transition or maintenance were included, except in Kampala and Wakiso, where we only took a 40% random sample of transition facilities. Using this process, a total of 275 facilities were included in the sample. Two case study facilities in the qualitative component of the parent study were added to the sample for a total of 277.

To measure the impact on service availability, the survey asked each facility’s in-charge whether the facility provided any of four HIV services: antiretroviral therapy (ART), HIV outreach, prevention of mother-to-child transmission (PMTCT), and HIV testing & counseling (HTC). In Uganda, health centre (HC) IIs typically provide only primary health services, including HTC, while most HC IIIs and nearly all HC IVs and Hospitals provide ART [24]. If the facility reported that they currently did not offer the service, the enumerator asked if they offered the service prior to the transition date. The transition date was established by the facility in-charge, if facility reported transition. If the facility reported maintenance, a fixed reference date of October 1, 2016 was used.

Facility in-charge respondents were also asked to report on their perceptions of changes in access to and quality of HIV services at their facility since the transition date. As these questions rely on self-report, they measure the in-charges’ of general access and quality to HIV and non-HIV related services. We compare responses on discontinuation and changes in quality and access across facilities reporting transition and maintenance using weighted Chi-square tests. Analysis of the facility survey data accounts for survey design using sampling weights, clustering at the district level, stratification, and finite population correction. However, many contingency tables are sparse, with fewer than 5 cases in each cell, making the chi-square test unreliable. Therefore, we also use an unweighted Fisher’s exact test as a sensitivity analysis.

The study was approved by the institutional review board of the Johns Hopkins Bloomberg School of Public Health (00007208). Local ethical approval was provided by the Uganda National Council of Science and Technology’s Research Ethics Committee (SS 4263).

### Service delivery data from DHIS2

We used the Health Management Information System (HMIS) obtained from the Uganda ministry of health’s district health information system (DHIS2) to examine changes in service utilization. We extracted data for the period October 2013 to December 2017 for all facilities reported by the USAID mission in Uganda to have had PEPFAR support in 2014. We selected HIV indicators that reflect each of the 90-90-90 goals [2]: HIV testing and counseling (“HTC”); numbers of patients currently on antiretroviral therapy (“Current on ART”); and retention in care on first-line ART at 12 months (“Retention”), which is a proxy for virologic suppression.

The DHIS2 dataset is increasingly being used for monitoring and evaluation of health services in Uganda and other LMICs, despite its known limitations. While numerous studies have noted quality issues in HMIS/DHIS2 datasets, including problems with completeness and accuracy [25–29], a recent study [27] shows that data quality in Uganda had improved just prior to the baseline period. Starting in 2015, PEPFAR began using DHIS2 data as the primary source for monitoring and reporting of HIV indicators in Uganda. There has also been some limited use of HMIS/DHIS2 data for evaluation of HIV programs in the peer-reviewed literature. In Uganda, Luboga et al. (2016) used district-level HMIS records to assess the impact of HIV scale-up on non-HIV care [30]. Other studies have tended to use facility registries or HMIS reporting forms [31–34] that feed into DHIS2.

We restricted our analysis to facilities that reported to the DHIS2 system at least two times during the anticipated baseline (October 2013 to June 2016) and post-transition (July 2016 to December 2017) periods. As most facilities are either fair-to-good reporters (>80% complete) or very poor reporters (<10%), a more stringent criterion (e.g. 3 reports) would change the composition of facilities only a little. In the facility survey sample, 208 of 226 (92%) could be included in the analysis of HTC, and 15/18 facilities excluded from the analysis for HTC were PFP facilities. Out of 166 facilities that reported providing ART, 139 (84%) could be included in the analysis of Current on ART and Retention (Table B in S1 File). Excluded facilities were disproportionately small and privately-owned. We performed a minimal data cleaning with the goal of removing out-of-range data values that could bias the analysis. Changes in the way Retention was reported in July 2015 resulted in a large proportion of out-of-bounds data (i.e. <1% or >100% Retention) that we excluded. The DHIS2 data cleaning process is described in greater detail in Table C in S1 File. There is no distinction between missing and null value in DHIS2. We imputed “0” for counts of HTC and Current on ART that are missing on a HMIS report that was submitted to DHIS2, but excluded missing for Retention on ART. Reporting rates for facilities in the survey sample are reported in Fig A-C in S1 File.

To evaluate the impact of transitioning from PEPFAR support, we constructed regression models to quantify the trends (i.e. linear slopes) in HIV services prior to and after October 2016 (the median transition date reported), separately for transition and maintenance facilities. Specifically, the models included time, a linear spline term for time to allow a change in linear slope post October 2016, an indicator for facility type (transition vs. maintenance) and the interaction between the facility type indicator and the time variables. Similar to the commonly used “difference-in-difference” approach [35], we computed the difference in the change in the linear slopes prior to and after October 2016 for the transition and maintenance facilities as a measure of the impact of transition. Using slopes rather than levels, we relax the assumption of parallel baseline trends.

The analysis described above used negative binomial regression models for the HTC and Current on ART variables since these are both count variables, with a possibility of overdispersion relative to the Poisson distribution. For Retention on ART, expressed as a proportion between 0 and 1, linear regression models were used. As a sensitivity analysis to the assumption of normally distributed residuals, we repeated the analysis by generating confidence intervals and hypothesis tests based on a non-parametric bootstrap procedure containing 1,000 bootstrap samples, where facilities were sampled with replacement.

To account for the correlation in HIV services over time within the same facility, random intercepts defined for each facility were included in the models and a Huber-White sandwich estimator was used to estimate standard errors for all regression coefficients. Dummy variables for month were included in the analysis of HTC to account for observed seasonal variation. In all models, we adjusted for facility level and ownership (public, PFP, private not for-profit [PNFP]).

We first fitted the models using only the subset of DHIS2-reporting facilities that are in our facility survey sample. In our survey sample, 208 facilities (of which 188 transition and 20 maintenance) have enough data for analysis of HTC as well as 139 for Current on ART and 138 for Retention (of which 11 are maintenance for both). Given the small number of facilities—particularly maintenance facilities—in our survey sample, we repeat the analysis of using all available DHIS2 data for facilities identified as PEPFAR-supported in FY2014. Rather than using facility-reported transition status, which is unavailable for facilities not in our survey sample, we use PEPFAR’s official transition intentions for each facility. PEPFAR GP transition intentions agree poorly with the transition status reported by facilities themselves (Table A in S1 File). Therefore, we consider the analysis using the full sample as an intention to treat (ITT) analysis. A total of 989 maintenance and transition facilities have enough data for analysis of HTC (of which 404 are intended for maintenance), 482 for analysis of Current on ART (354 maintenance), and 477 for Retention (353 maintenance). Otherwise, the secondary analysis is the same as described previously. We do not use weights or clustering in the analysis of DHIS2 data. All analyses were conducted using Stata 15 [36].

## Results

### Facility survey descriptive statistics

Enumerators were able to complete surveys at 262 facilities. Of the 15 facilities that could not be surveyed, nine had closed permanently, two were closed for construction, two facilities were identified as duplicate records, one refused to participate in the survey, and one was not accessible on account of hazardous road conditions. Of the 262 facilities surveyed, 206 reported having been transitioned, 20 reported continuing to receive PEPFAR support, and 36 claimed to have had no PEPFAR support within the past 3 years. Contrary to expectations, there was only 54% agreement between PEPFAR GP transition intentions and self-reported transition status in our facility survey (Table A in S1 File).

From follow-up interviews with IPs and USAID, we determined that many transitioned facilities were experiencing a break in support between IPs due to contract turnover lasting for about 9–12 months. As these facilities reported similar processes and impacts as those that were genuinely transitioned, we have included them as transition facilities in this analysis. We exclude the 36 facilities that report no past PEPFAR support from the analysis.

The characteristics of the facilities surveyed are presented in Table 1. Transition facilities were, more likely to be private for-profit facilities (23.9% vs. 3.1%), and less likely to be PNFP facilities (14.6% vs. 27.7%). There were also fewer hospitals and more health centre III facilities reporting transition.

**Table 1:** Facility characteristics in survey sample.

	Transition		Maintenance	
	No.	Weighted %	No.	Weighted %
**Facility Level**				
*HC II or Clinic*	50	32.4%	6	37.7%
*HC III*	133	57.7%	10	27.7%
*HC IV*	14	6.2%	1	5.2%
*Hospital*	9	3.7%	3	19.4%
**Facility Ownership**				
*Public*	145	61.5%	14	69.1%
*Private not for-profit*	29	14.6%	5	27.7%
*Private for-profit*	32	23.9%	1	3.1%
**Transition Wave**				
*2013–2014*	18	10.9%	n/a	n/a
*2015–2016*	118	60.1%	n/a	n/a
*Jan–May 2017*	70	29.0%	n/a	n/a
**Services Available**				
*Provides ART*	149	61.1%	15	69.3%
**Total**	206	100%	20	100%

n/a–not applicable.

Many facilities reported transition in 2013–2014, prior to the formal start of the GP in October 2015. However, these facilities were identified by USAID as part of the GP. Major transition activity did not take place until 2015–2016, and by October 2016, half of the transitions in our facility sample had taken place.

Among transitioned facilities, 70% report having been informed of transition, mostly by departing IPs.; facilities were informed an average of 3 months in advance of transition, and only 40% of transitioned facilities reported having a strategy to cope with transition (not shown in Table 1)

### Results from facility survey

Table 2 summarizes the reported changes in access and quality of HIV care by facility in-charge respondents. Respondents in transition facilities were more likely to report worsening patient access for HIV services than in-charges at maintenance facilities (43.5% vs. 3.1%). They are also more likely to report that the quality of HIV care was deteriorating than maintenance facility in-charges (35.6% vs. 0%). Overall, maintenance facilities were positive about access and quality of HIV care, with 81% and 92% reporting “Better” or “Much better” for access and quality, respectively.

**Table 2:** In-charge reported change in access and quality of care.

Change	Access of HIV Services for Average Patient^1^		Overall Quality of HIV Services^1^	
	Transition	Maintenance	Transition	Maintenance
**Worse/Much Worse**	43.5%	3.1%	35.6%	0.0%
**Same**	36.0%	10.3%	42.0%	3.1%
**Better/Much Better**	18.4%	81.4%	21.2%	91.7%
**Number of facilities**	204	19	204	19
**Weighted X2 p-value**	<0.001		<0.001	
**Fisher’s Exact Test** **p-value**	<0.001		<0.001	

^1^Category of “Don’t know/Not Applicable” excluded. Percentages may not sum to 100% as a result.

Respondents reported substantial discontinuation of only one HIV service—HIV outreach (not shown). Among facilities that provided outreach prior to transition, 51.6% of transition facilities discontinued outreach, compared to only 4.1% of maintenance facilities (Weighted Χ^2^ p-value: p<0.001). Discontinuation of HTC, ART, and PMTCT were reported at 2.4%, 1.3%, and 4.2% of transition facilities, respectively, compared to 0% of maintenance facilities (not significant).

### Outcomes from DHIS2 data: Facility survey sample

**Fig 1:**
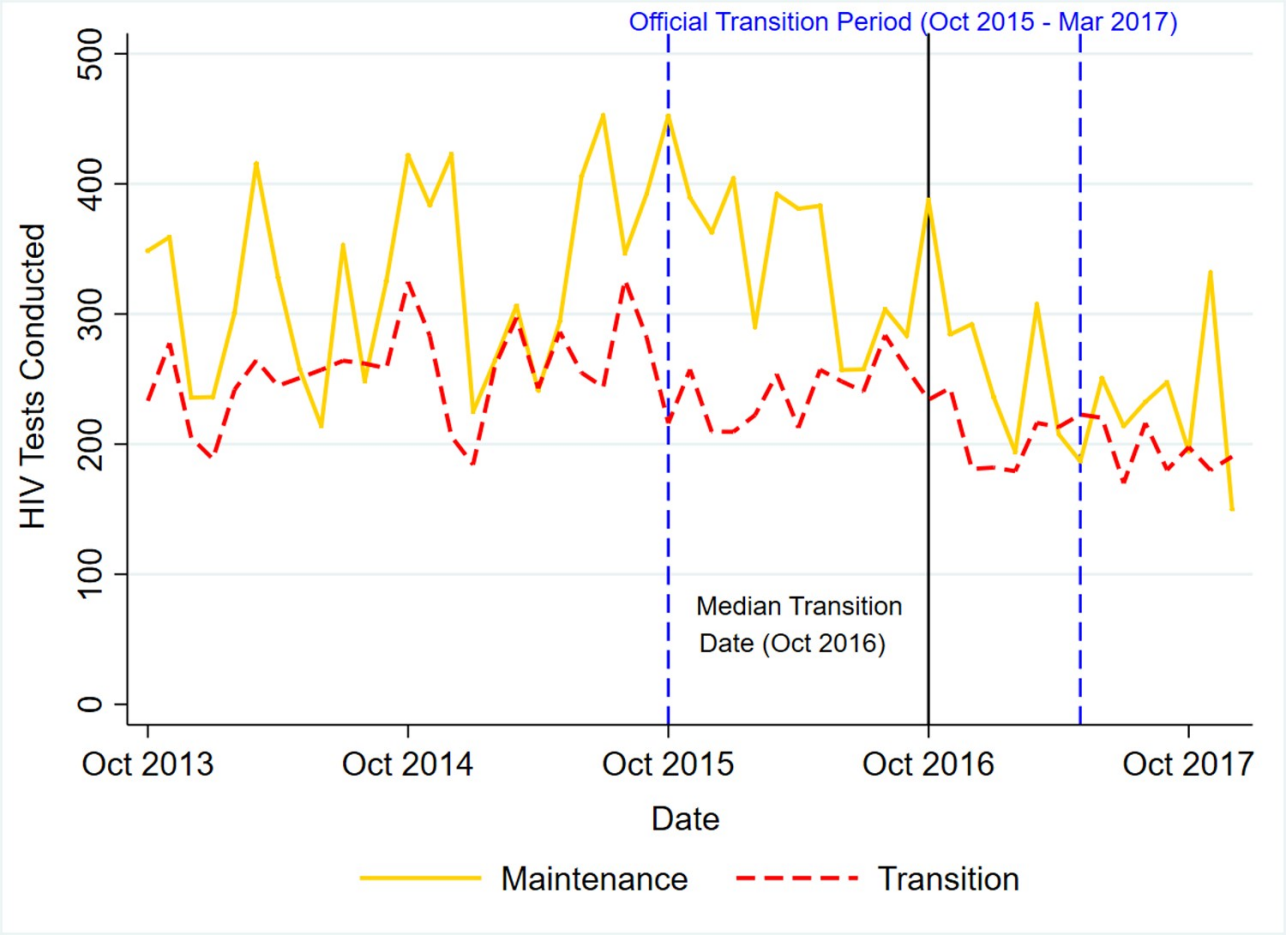
DHIS2 trends in HIV testing & couseling by transition status.

**Fig 2:**
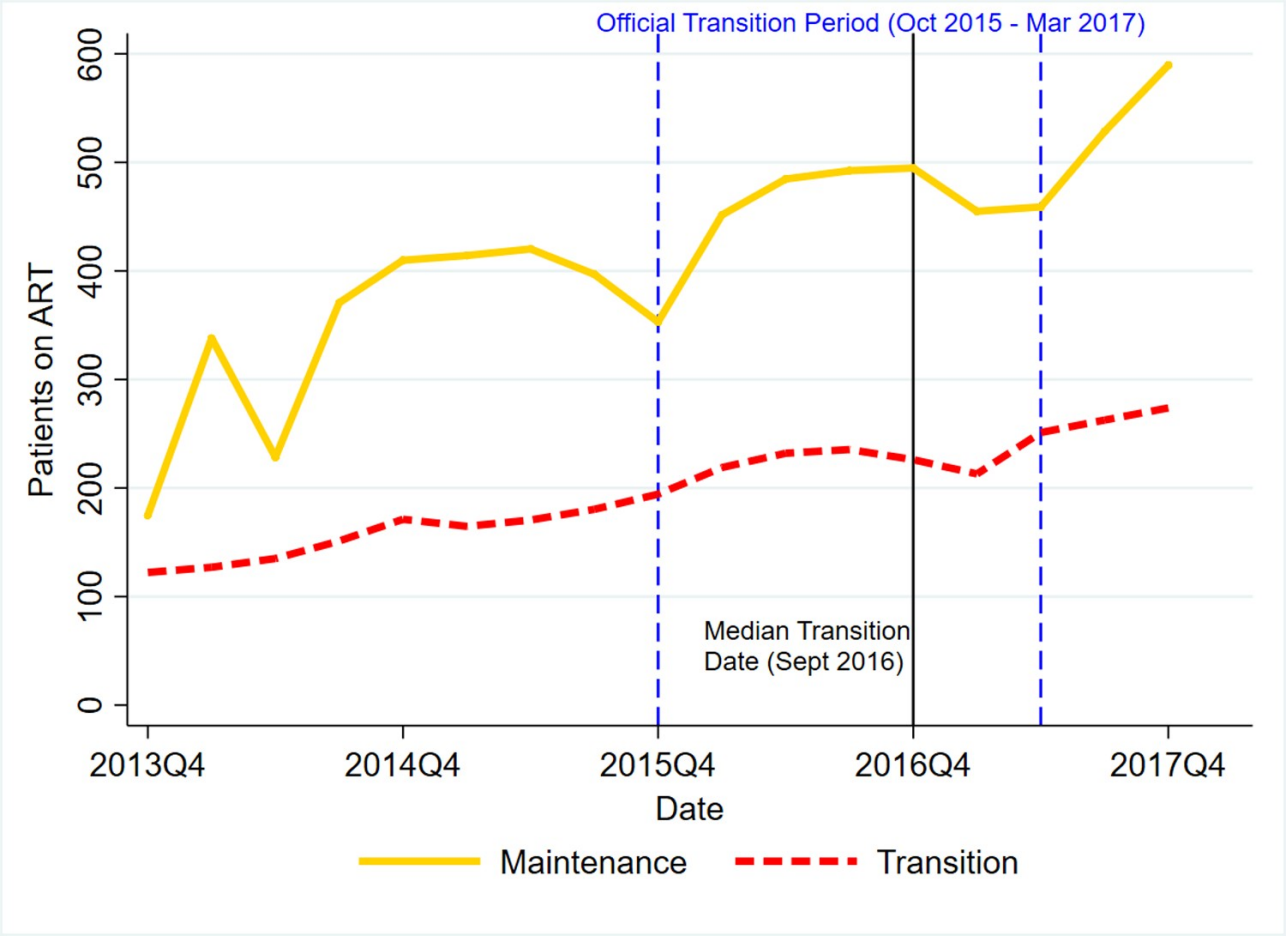
DHIS2 trends in current on ART by transition status.

**Fig 3:**
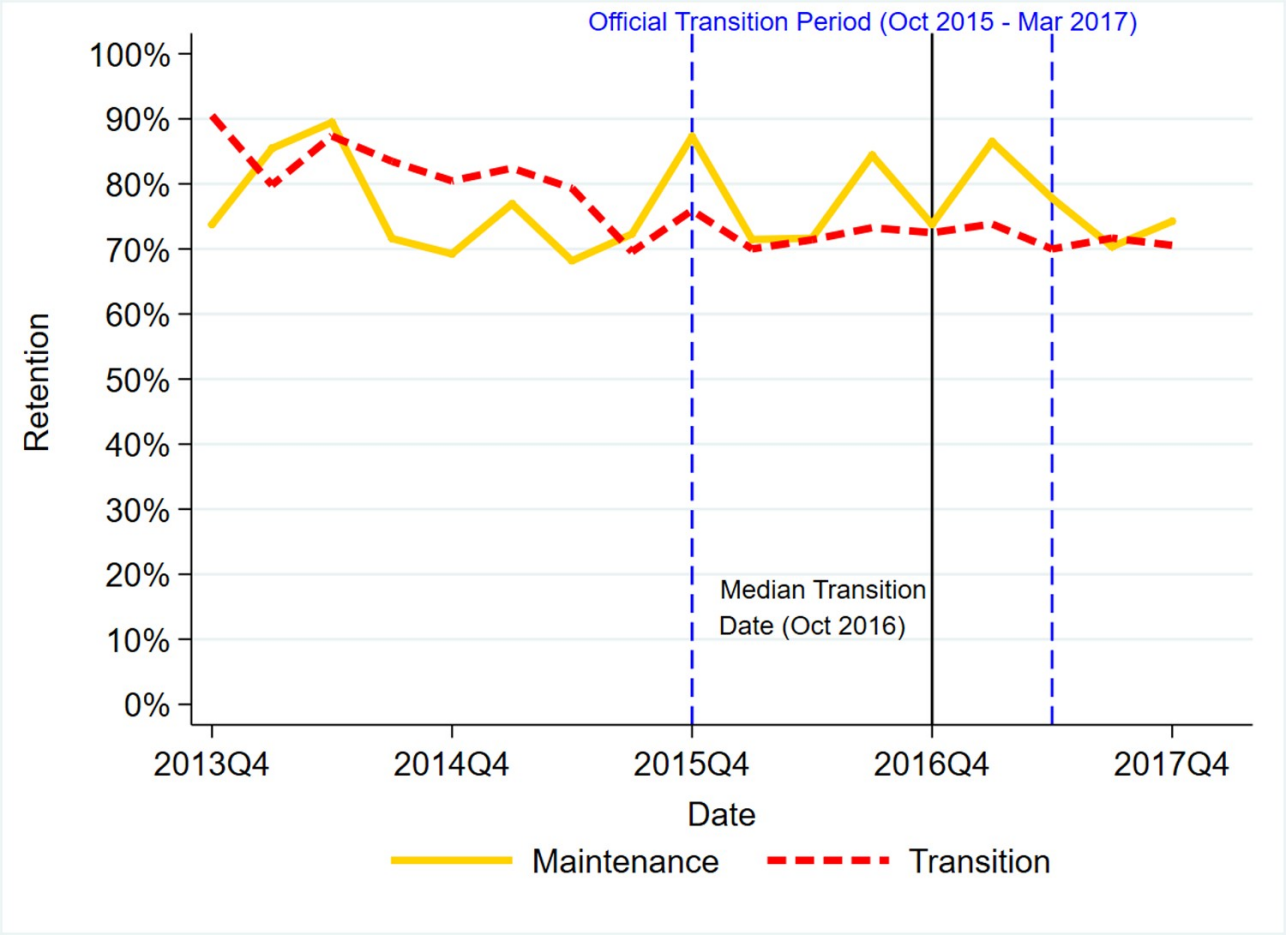
DHIS2 trends in 12-month retention on first-line ART by transition status.

Figs 1–3 present trends for transition and maintenance facilities in HTC, Current on ART, and Retention, respectively.

Table 3 presents the results of the DHIS2 regression analysis, including a calculation of the pre- and post-Oct 2016 slopes and the difference-in-difference in slopes. For HTC, testing was declining at -0.3% per month in both transition and maintenance, but the rate of decline accelerated to -1.2% per month for maintenance and -2.6% per month for transition, giving a difference-in-difference in slopes of -1.5% per month (95% C.I.: -7.3%, 4.3%; p = 0.618).

**Table 3:** Adjusted trends in service delivery–DHIS2 (survey sample).

Service Indicator	Frequency	Maintenance		Transition		Impact of Transition on Slopes (95% C.I.)^1^	Robust P-value	N Facilities (Maintenance, Transition)
		Pre Slope	Post Slope	Pre Slope	Post Slope			
**HTC**^**2**^	Monthly	-0.3%	-1.2%	-0.3%	-2.6%	-1.5% (-7.3%, 4.3%)	0.618	208 (20, 188)
**Current on ART**	Quarterly	8.7%	-1.4%	8.2%	4.5%	6.3% (-1.0%, 13.7%)	0.093	139 (11, 128)
**Retention**	Quarterly	-0.8%	0.1%	-1.4%	-0.2%	-0.3% (-2.5%, 3.2%)	0.832	138 (11, 127)

^1^ The difference in the change in linear slopes from pre- to post-October 2016 for transition minus that of maintenance.

^2^ Also adjusted for seasonal variation.

For the number of patients currently on ART, maintenance and transition facilities were increasing at a rate of 8.7% and 8.2% per quarter, respectively, prior to October 2016. After October 2016, the slope turned negative to -1.4% per quarter for maintenance and remained positive at 4.5% per quarter for transition, leading to a difference-in-difference in trend of 6.3% per quarter (95% C.I.: -1.0%, 13.7%, p = 0.093).

For Retention on ART, the negative trends in both maintenance and transition flattened out after transition. The difference-in-difference in slopes is -0.3% per quarter (95% C.I. 2.5%, 3.2%; p = 0.832). Using bootstrap resampling for Retention does not alter the interpretation. Full models are presented in Table D in S1 File.

### DHIS2 full sample: Intention to treat analysis

Repeating the primary analysis with all available PEPFAR-supported facilities in DHIS2 and using official PEPFAR transition classifications (“ITT Analysis”), the findings are slightly different (Table 4). Facilities that PEPFAR intended to transition had a significant positive difference-in-difference in slopes in HTC of 3.1% per month (95% C.I. 1.6%, 4.5%, p<0.001). The impact of transition on trends for Current on ART and Retention were not significant. Full model results are included in Table E in S1 File.

**Table 4:** Adjusted trends in service delivery (full sample, ITT analysis).

Service Indicator	Frequency	Maintenance		Transition		Impact of Transition on Slopes (95% C.I.)^1^	Robust P-value	N Facilities (Maintenance, Transition)
		Pre Slope	Post Slope	Pre Slope	Post Slope			
**HTC**^**2**^	Monthly	0.3%	-0.3%	-0.8%	1.7%	**3.1%** **(1.6%, 4.5%)**	**<0.001**	989 (585, 404)
**Current on ART**	Quarterly	8.3%	5.5%	8.2%	6.6%	1.2% (-2.4%, 5.0%)	0.518	482 (128, 354)
**Retention**	Quarterly	-0.9%	-0.8%	-0.5%	-1.2%	-0.8% (-2.1%, 0.5%)	0.240	477 (126, 353)

^1^ The difference in the change in linear slopes from pre- to post-October 2016 for transition minus that of maintenance.

^2^ Also adjusted for seasonal variation.

## Discussion

Facilities transitioned from PEPFAR support were far more likely to discontinue HIV outreach than facilities maintained on PEPFAR. HIV outreach is an important service linking facilities and communities. This study did not define outreach precisely, but it is likely to include bringing HIV testing services to populations that do not regularly seek care at facilities, particularly men and adolescents [37], as well as providing adherence counseling, defaulter tracing, and other efforts that have been demonstrated as successful in improving retention in care [38, 39].

Transition facility respondents were also more likely to report that patient access and quality of HIV care had declined than respondents in maintenance facilities. These findings offer reasons to be concerned about transition’s impact on HIV services. However, the DHIS2 data paints a different picture of transition. Transitioned facilities in our survey did not have significantly different changes in their trends for HIV service indicators compared to maintenance facilities. When including all available PEPFAR facilities in Uganda, facilities intended for transition did better in terms of HTC and did no worse for Current on ART or Retention.

Unrelated to transition, the decline in retention on first-line ART is a cause for concern. The decline is also reflected in nationwide UNAIDS estimates for Uganda, which show retention falling from 85% in 2014 to 78% in 2017 [40]. This study was not intended to explain the cause of the decline in retention, but it is possible that increased ART eligibility under “test and treat” has led to enrollment of relatively healthy people living with HIV, who may be less motivated to remain in care, as well as patients in remote rural areas, for whom retention tends to be lower [41]. However, it is also possible that our measure of facility-level retention underestimates patient-level retention by not adequately accounting for patients switching providers without referrals [42]. If switching is increasing over time, this may explain the decline in facility-based retention. The importance of retention in care is underlined by the emphasis on “treatment as prevention”. A simulation suggests that improving retention is critical to cost-effectively reducing HIV incidence in Uganda [43]. More research is needed on the causes of the decline and on possible interventions to improve retention.

This study has multiple limitations. First, the facility survey findings are based on self-report by health workers and, therefore, may be subject to response and recall bias. Survey respondents may have intended to portray transition from PEPFAR in a negative light in order to encourage policymakers to reinstate support. Transition facility respondents may also be more likely to recall negative outcomes and associate them with transition than respondents in maintenance facilities. Furthermore, the lack of national representativeness means that the findings from our survey sample may not be generalizable for all transition and maintenance facilities in Uganda.

The small size of our survey, particularly for maintenance facilities, makes our comparison unreliable and reduces power to detect diverging trends. For example, the 95% confidence intervals for Current on ART in the survey sample suggest a relative change in the number of patients on ART that is 4% lower or 67% higher in transition facilities after one year, compared to maintenance. We used the full set of PEPFAR data in DHIS2, which is three to four times larger than our facility survey sample, to increase power. However, this sample can only be considered as intention to treat, and, from our facility survey, there is poor agreement between facility-reported and PEPFAR intentions. Random error in our measurement of facility transition status in the ITT analysis would reduce estimates of the impact of transition towards zero.

The completeness and quality of DHIS2 data is known to be a limitation. In our facility survey, 92% of facilities have enough data for HTC but only 84% of facilities that report ART programs could be included in the analysis of Current on ART and Retention. While there are likely to be differences between reporting and non-reporting facilities, we consider it less likely (though possible) that reporting facilities experienced and responded to transition differently than non-reporting facilities.

Our efforts to clean DHIS2 data only removed the most extreme outliers and likely did little to improve the quality of the data. However, our findings would only be biased if the quality of data changed differentially between maintenance and transition facilities around the time of transition. This would be the case if the quality of data deteriorated in transition facilities and/or improved in maintenance following transition. Annual data supervision visits by PEPFAR IPs were meant to be continued as part of the package of “central support” retained by transition facilities, but at the time of writing we do not know if these visits have continued.

A further limitation is that we cannot draw causal conclusions from the DHIS2 findings. In order to consider the estimates from trend analysis as causal impacts, we need to assume independence of facilities and that selection of transition facilities does not depend on baseline trends in the baseline indicator. The first assumption is violated if patients are systematically switching between maintenance and transition facilities, which is possible in this context, even though it is not observed in the data. Furthermore, since some transition facilities were selected based on being “low-volume” for HIV indicators, the second assumption is also violated. Regression to the mean may cause facilities classified as “low-volume” to have faster growth in HIV services unrelated to transition.

Lastly, we have data for only 15 months after the midpoint of transition. In a forthcoming article from the parent study, we note that transition facilities reported loss of lay health workers, reductions in training, and a decline in the frequency of HIV supervision. These impacts on the health workforce may take time to translate into changes in HIV service volume. The respondents in our survey reporting declining access and quality of HIV services may have a perspective on changes that will affect provision of HIV services beyond our follow-up period. However, it is also possible that survey respondents are underestimating the resiliency of health facilities to cope with loss of PEPFAR support. Given these findings, we argue in favor of a cautiously optimistic approach towards transition of health facilities and call for further empirical research.

## Conclusion

Transition from PEPFAR is associated with a reported reduction in HIV outreach, access to HIV care, and quality of care. Although facility respondents reported concerns about declines in patient access and quality of HIV service provision, service delivery trends derived from DHIS2 do not show an immediate impact of transition. More follow-up is needed to determine if facilities transitioned from PEPFAR support continue to keep pace with maintenance facilities in terms of HIV service delivery. Attention also needs to be paid to the decline of retention on ART in Uganda.

## Supporting information

S1 File Supplemental file. (DOCX)
