# Supplementary material for: The impact of PEPFAR transition on HIV service delivery at health facilities in Uganda
Source: PLoS One. 2019 Oct 9;14(10):e0223426. doi: 10.1371/journal.pone.0223426 (PMC6785126; doi:10.1371/journal.pone.0223426)
Supplement: S1 File — (DOCX) [file pone.0223426.s001.docx]

**Supplemental File**

**Table A: Comparison of PEPFAR GP Transition Intentions and Facility-Reported Transition Status**

| **PEPFAR Official Transition Designation** | **Facility-Reported Transition Status** | | |
| --- | --- | --- | --- |
|  | **Transition** | **Maintenance** | **No Support** |
| **Transition** | **136**  **(51.9%)** | 16  (6.1%) | 34  (13.0%) |
| **Maintenance** | 68  (26.0%) | **6**  **(2.3%)** | 2  (0.8%) |

Footnotes: PEPFAR, President’s Emergency Plan for AIDS Relief. Cells in bold indicate agreement.

**Assessment of Case-Level Missing:**

Missing data in the DHIS2 analysis comes in two forms: case-level missing for facilities that are either not in DHIS2 or do not have the necessary 2 reports in pre and post-transition periods to be included in the analysis, and item-level missing. We assume that item level missing is at random, and our mixed effects models are robust to missing at random. Case level missing is problematic, as excluded facilities may differ significantly from those with sufficient data to be included in the analysis. In supplemental table B we compare the characteristics for facilities included and excluded from the analysis.

Nearly all of the 8% of missing facilities for HTC from the facility survey sample are private for-profit facilities, all of which reported transition. These facilities tend to be small, typically Health Centre IIs or Clinics, in the Ugandan system. As the transition category in the dataset is lacking in private for-profit facilities, the findings can likely be generalized only to the public and private not-for-profit sectors.

For ART outcomes, there are 164 facilities (159 transition, 15 maintenance) that reported providing ART before transition through the survey. There were an additional 2 facilities that report ART outcomes in DHIS2 but not in the survey. They are counted in the denominator here. Among them, 84% have enough data for ART in DHIS2 (85% for transition and 73% for maintenance). Private for-profit and private not for profit facilities are more likely to be missing than public facilities. Lower level HC IIs & IIIs account for a disproportionate share of the missing. While HC IIs lack clinical officers and are therefore not allowed to provide ART in the public system, private for-profit clinics are classed along with HC IIs, and can provide ART if appropriately staffed and accredited.

Within the full sample, we focus on the HTC outcomes. There is no objective way to determine which facilities provide ART and are therefore missing if they do not report to DHIS2 or were not in our survey. However, all PEPFAR supported facilities are supposed to provide and report HTC. Out of 1,153 facilities designated for either transition or maintenance, 57 facilities are not in DHIS2 at all and a further 90 facilities lack enough data in DHIS2. Of the remaining 1,006 facilities, 17 lack covariate data (level, ownership) used in fitting models. Unlike in the facility survey sample, covariate information was not directly accessed and can be missing from DHIS2. Therefore, in Table B, we compared facilities from the 989 (out of 1,006) included in the analysis to the 70 facilities that are not in DHIS2 or lack enough data points but do have covariate data available. From the data available, low reporting by private for-profit and small facilities are responsible for the bulk of the missing data. Though not negligible, these small, private for-profit facilities contribute only a small fraction of HIV services in Uganda. Therefore, our findings are representative of facilities that provide most of the HIV care in the country.

**Table B:** **Characteristics of Facilities Included and Excluded from the Analysis of DHIS2 Outcomes**

|  | **Facility Survey Sample**  **(N= 226)** | | **Intention to Treat Sample**  **(N = 1,069 with covariate data)** | |
| --- | --- | --- | --- | --- |
|  | **With Enough Data** | **Either not in DHIS2 or Not Enough Data** | **With Enough Data** | **Either not in DHIS2 or Not Enough Data** |
| **N For HTC** | 208 | 18 | 989 | 70 |
| **% of Total** | 92% | 8% | 86% | 14% |
| **% of Transition^1^** | 91% | 9% | 90% | 10% |
| **% of Maintenance^1^** | 100% | 0% | 99.5% | 0.5% |
| **Ownership (% Column)** |  |  |  |  |
| *Public* | 158 (76%) | 1 | 738 (75%) | 13 (19%) |
| *Private Not for Profit* | 32 (15%) | 2 | 183 (19%) | 13 (18%) |
| *Private for Profit* | 18 (9%) | 15 | 68 (7%) | 44 (63%) |
| **Level (%)** |  |  |  |  |
| *HC II or Clinic* | 42 (20%) | 14 | 460 (47%) | 64 (91%) |
| *Health Centre III* | 139 (67%) | 4 | 438 (44%) | 6 (9%) |
| *Health Centre IV* | 15 (7%) | 0 | 56 (6%) | 0 |
| *Hospital* | 12 (6%) | 0 | 35 (4%) | 0 |
| **N for ART^2^** | 139 | 27 | 482 | n/a |
| **% of Total^3^** | 84% | 16% | n/a | n/a |
| **% of Transition^1, 3^** | 128 (85%) | 23 (15%) | n/a | n/a |
| **% of Maintenance^1, 3^** | 11 (73%) | 4 (27%) | n/a | n/a |
| **Ownership (% of ART Facilities in Column)** |  |  | n/a | n/a |
| *Public* | 122 (88%) | 15 (56%) | n/a | n/a |
| *Private Not for Profit* | 16 (12%) | 10 (37%) | n/a | n/a |
| *Private for Profit* | 1 (1%) | 2 (7%) | n/a | n/a |
| **Level (% of ART Facilities in Column)** |  |  | n/a | n/a |
| *HC II or Clinic* | 4 (3%) | 7 (26%) | n/a | n/a |
| *Health Centre III* | 110 (79%) | 19 (70%) | n/a | n/a |
| *Health Centre IV* | 14 (10%) | 0 (0%) | n/a | n/a |
| *Hospital* | 11 (8%) | 1 (4%) | n/a | n/a |

^1^For the facility survey, transition status is self-report, while in the ITT sample, it is official PEPFAR transition intentions.

^2^Using Current on ART as a proxy for both ART outcomes (Current on ART, Retention on ART)

^3^There are 166 facilities that report providing ART in either the facility survey or DHIS2 (151 transition, 15 maintenance). Provision of ART is not known for facilities that do not report to DHIS2 and are not included in the facility survey.

**Assessment of Item Level Missing - Reporting Rates:**

There is item-level missing due to missing or flagged data during some reporting periods. Our models are robust to missing under the missing at random assumption, which requires that missing be independent of the outcome at the level of the facility. If this assumption is met, changing reporting rates will not bias the analysis. However, this assumption could be violated if changes in reporting coincided with changes in the outcome.

Supplemental figures A–C show the reporting rates for indicators to DHIS2 for facilities in our facility survey sample. Reporting rates are very high among both transition and maintenance facilities. Reporting for HTC improved during the period prior to transition and remained steady afterwards. The dip in reporting in December 2017 is due to late reporting by some facilities. Data extraction took place in late January of 2018.

**Figure A –** **Reporting Rates for HIV Testing & Counseling in the Facility Survey Sample**

**
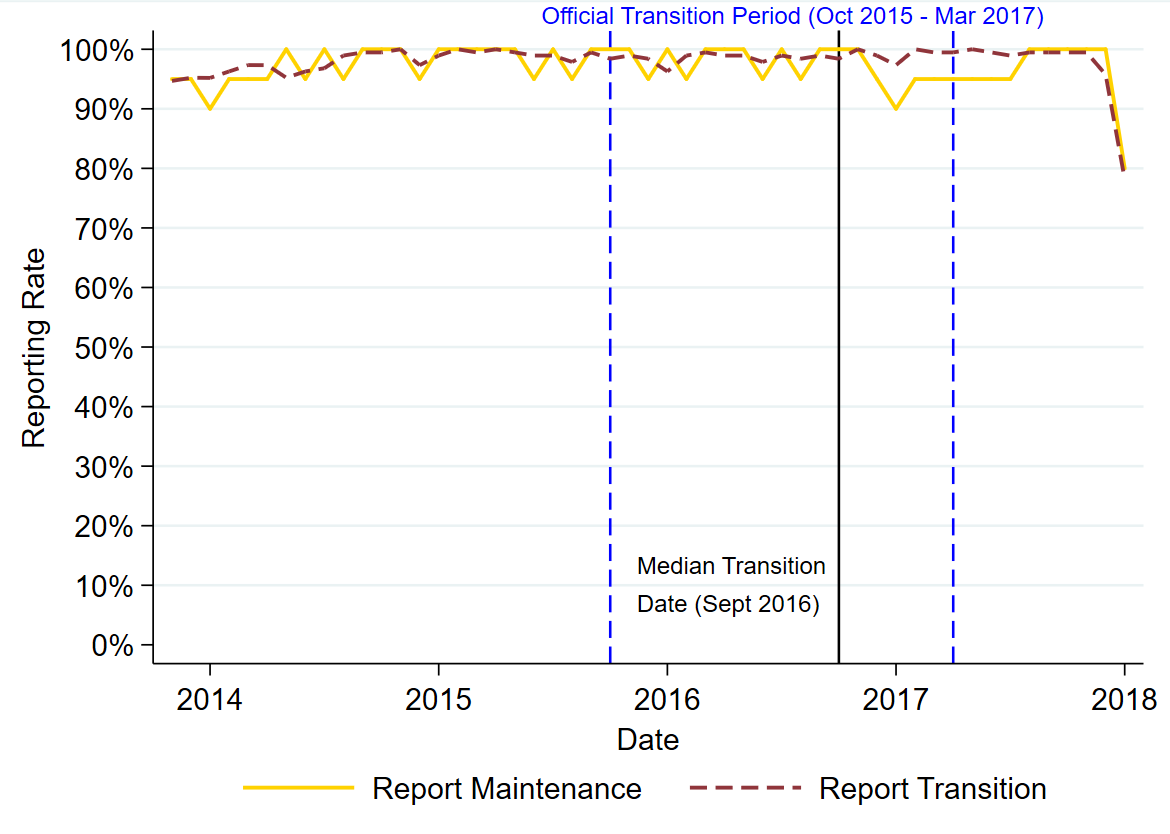
**

**Figure** **B: Reporting Rates for Current on ART in the Facility Survey Sample**

**
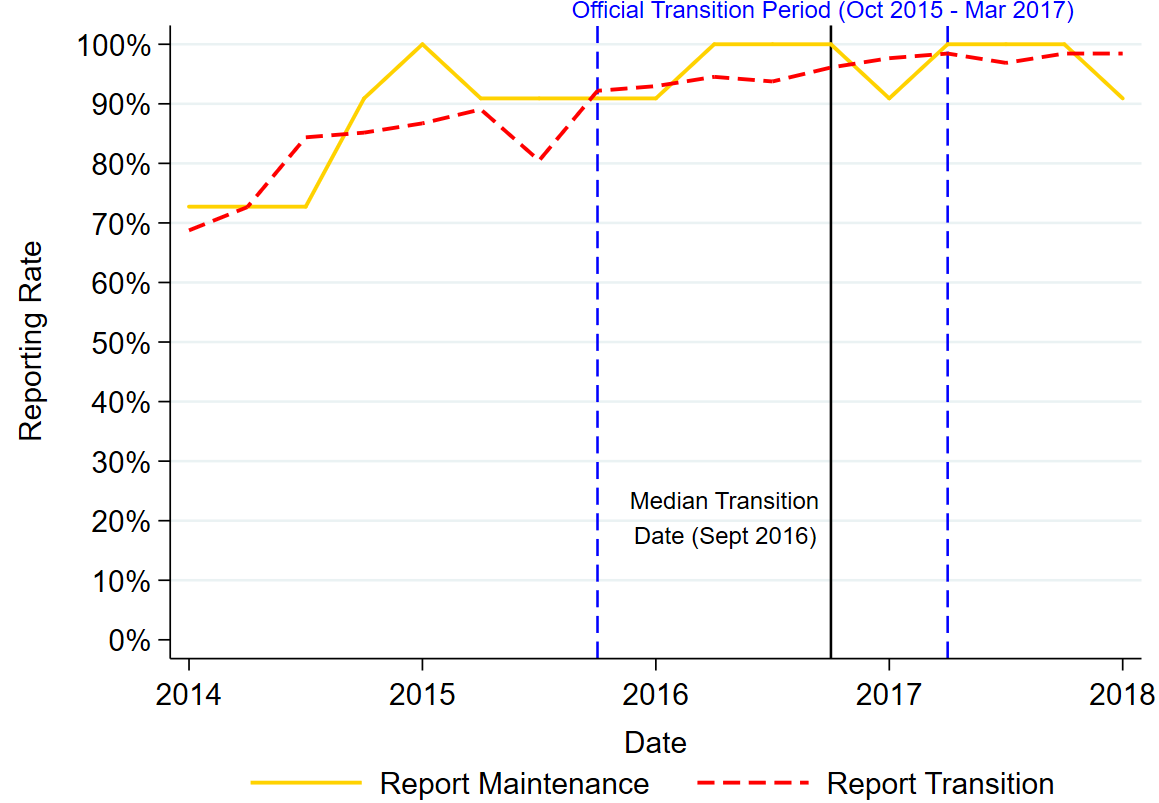
**

Reports with missing cells for HTC and Current on ART were imputed to zero. Since we imputed “0” for missing on valid reports, the high reporting rates reflect whether a valid report was submitted. However, we did not impute for Retention on ART, and we flagged a 11-13% (Table C) of values (i.e. set them to missing) due to out-of-range data. The reporting rates are lower for Retention on ART than Current on ART, especially after the change in reporting forms in July 2015. However, reporting rates for maintenance and transition track one another.

**Figure C:** **Reporting Rates for 12-month Retention on ART in the Facility Survey Sample**


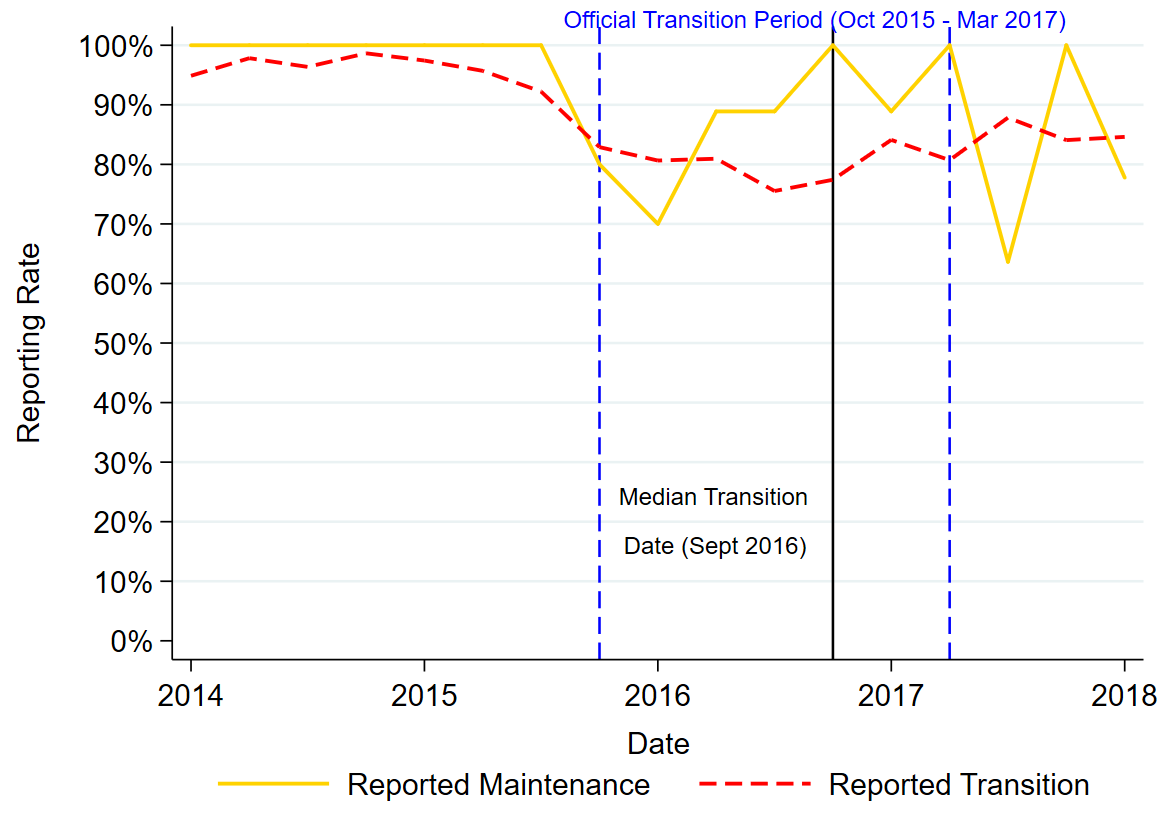


**Table C: Observations Excluded During Data Cleaning**

| **Analysis Type** |  | **HTC** | | **Current on ART** | | **Retention on ART** | |
| --- | --- | --- | --- | --- | --- | --- | --- |
|  |  | **N** | **%** | **N** | **%** | **N** | **%** |
| **Facility Survey** | Excluded | 3 | 0.03% | 0 | 0% | 215 | 13.2% |
|  | Total | 10,339 |  | 2,220 |  | 1632 |  |
| **ITT Analysis** | Excluded | 29 | 0.06% | 4 | 0.05% | 702 | 11.3% |
|  | Total | 49,216 |  | 7,858 |  | 6,207 |  |

Footnote: ART, antiretroviral therapy; HTC, HIV testing & counseling; ITT, intention to treat.

**Table D: Full Regression Models (Facility Survey Sample)**

|  |  | **HTC** | **Current on ART** | **Retention on ART** | **Retention on ART** |
| --- | --- | --- | --- | --- | --- |
| **Model Type** | | **Neg. Binomial** | **Neg. Binomial** | **Gaussian** | **Gaussian** |
|  |  | **Beta** | **Beta** | **Percent** | **Percent** |
| **Covariates** | | **(Robust 95% C.I.)** | **(Robust 95% C.I.)** | **(Robust 95% C.I.)** | **(Bootstrap 95% C.I.)** |
| **Level:** | *HC III vs. HC II* | **1.813***** | -0.165 | -0.009 | -0.009 |
|  |  | **(1.042, 2.585)** | (-1.648, 1.318) | (-0.129, 0.111) | (-0.161, 0.143) |
|  | *HC IV or Hospital vs. HC II* | **2.808***** | **2.020*** | -0.052 | -0.052 |
|  |  | **(1.999, 3.616)** | **(0.468, 3.572)** | (-0.179, 0.074) | (-0.211, 0.106) |
| **Ownership** | *PNFP vs. Public* | -0.200 | **-0.749*** | 0.050 | 0.050 |
|  |  | (-0.67, 0.27) | **(-1.479, -0.019)** | (-0.012, 0.113) | (-0.014, 0.115) |
|  | *PFP vs. Public* | -0.496 | **-1.440***** | -0.001 | -0.001 |
|  |  | (-1.573, 0.581) | **(-1.664, -1.216)** | (-0.026, 0.024) | (-0.026, 0.024) |
| **Trends for Maintenance** | *Pre-Oct 2016* | -0.003 | **0.087***** | -0.008 | -0.008 |
|  |  | (-0.025, 0.018) | **(0.047, 0.127)** | (-0.018, 0.001) | (-0.018, 0.001) |
|  | **Δ** *Post-Oct 2016* | -0.008 | **-0.101**** | 0.010 | 0.010 |
|  |  | (-0.065, 0.049) | **(-0.171, -0.03)** | (-0.017, 0.036) | (-0.019, 0.038) |
| **Δ Trend for Transition** | *Pre-Oct 2016* | 0.000 | -0.005 | -0.006 | -0.006 |
|  |  | (-0.021, 0.022) | (-0.046, 0.037) | (-0.015, 0.003) | (-0.015, 0.004) |
|  | **Δ** *Post-Oct 2016* | -0.015 | 0.063 | 0.003 | 0.003 |
|  |  | (-0.073, 0.043) | (-0.010, 0.137) | (-0.025, 0.032) | (-0.027, 0.033) |
| **Month** | *February* | 0.056 |  |  |  |
| *(Reference = January)* |  | (-0.047, 0.159) |  |  |  |
|  | *March* | **0.219**** |  |  |  |
|  |  | **(0.08, 0.358)** |  |  |  |
|  | *April* | 0.122 |  |  |  |
|  |  | (-0.03, 0.275) |  |  |  |
|  | *May* | **0.218***** |  |  |  |
|  |  | **(0.101, 0.336)** |  |  |  |
|  | *June* | **0.206***** |  |  |  |
|  |  | **(0.098, 0.315)** |  |  |  |
|  | *July* | 0.110 |  |  |  |
|  |  | (-0.014, 0.234) |  |  |  |
|  | *August* | **0.309***** |  |  |  |
|  |  | **(0.201, 0.418)** |  |  |  |
|  | *September* | **0.241***** |  |  |  |
|  |  | **(0.124, 0.359)** |  |  |  |
|  | *October* | **0.193***** |  |  |  |
|  |  | **(0.082, 0.304)** |  |  |  |
|  | *November* | **0.267***** |  |  |  |
|  |  | **(0.153, 0.381)** |  |  |  |
|  | *December* | 0.016 |  |  |  |
|  |  | (-0.082, 0.114) |  |  |  |
| **Constant** |  | **3.333***** | **3.467***** | **0.902***** | **0.902***** |
|  |  | **(2.514, 4.152)** | **(1.984, 4.951)** | **(0.778, 1.027)** | **(0.745, 1.060)** |
| N | *Observations* | 10,385 | 2,220 | 1,417 | 1,417 |
|  | *Facilities* | 208 | 139 | 138 | 138 |
|  | *Transition* | 188 | 128 | 127 | 127 |
|  | *Maintenance* | 20 | 11 | 11 | 11 |

*p<0.05, **p<0.01, ***p<0.001

**Table E: Full Models for ITT Sample**

|  |  | **HTC** | **Current on ART** | **Retention on ART** | **Retention on ART** |
| --- | --- | --- | --- | --- | --- |
| **Model Type** | | **Neg. Binomial** | **Neg. Binomial** | **Gaussian** | **Gaussian** |
|  |  | **Beta** | **Beta** | **Percent** | **Percent** |
| **Covariates** | | **(Robust 95% C.I.)** | **(Robust 95% C.I.)** | **(Robust 95% C.I.)** | **(Bootstrap 95% C.I.)** |
| **Level:** | *HC III vs. HC II* | **2.474***** | **0.763***** | -0.009 | -0.009 |
|  |  | **(2.255, 2.694)** | **(0.335, 1.191)** | (-0.048, 0.031) | (-0.048, 0.031) |
|  | *HC IV vs. HC II* | **3.359***** | **2.516***** | **-0.053*** | **-0.053*** |
|  |  | **(3.053, 3.666)** | **(2.034, 2.998)** | **(-0.1, -0.006)** | **(-0.102, -0.005)** |
|  | *Hospital vs. HC II* | **3.187***** | **3.263***** | -0.012 | -0.012 |
|  |  | **(2.552, 3.823)** | **(2.701, 3.824)** | (-0.066, 0.042) | (-0.068, 0.043) |
| **Ownership** | *PNFP vs. Public* | 0.145 | **-0.592***** | -0.016 | -0.016 |
|  |  | (-0.070, 0.360) | **(-0.874, -0.311)** | (-0.045, 0.013) | (-0.046, 0.014) |
|  | *PFP vs. Public* | **0.794**** | -0.082 | **0.066***** | **0.066**** |
|  |  | **(0.321, 1.266)** | (-1.105, 0.942) | **(0.030, 0.101)** | **(0.024, 0.107)** |
| **Trends for Maintenance** | *Pre Oct 2016* | **0.004**** | **0.080***** | **-0.009***** | **-0.009***** |
|  |  | **(0.002, 0.006)** | **(0.073, 0.086)** | **(-0.011, -0.007)** | **(-0.011, -0.007)** |
|  | *Post Oct 2016* | -0.007 | **-0.027***** | 0.004 | 0.004 |
|  |  | (-0.015, 0.001) | **(-0.039, -0.014)** | (-0.002, 0.010) | (-0.002, 0.010) |
| **Δ Trend for Transition** | *Pre Oct 2016* | **-0.010***** | -0.001 | 0.001 | 0.001 |
|  |  | **(-0.015, -0.005)** | (-0.015, 0.013) | (-0.003, 0.004) | (-0.003, 0.004) |
| **Δ in Δ in Trend for Transition** | *Post Oct 2016* | **0.031***** | 0.012 | -0.008 | -0.008 |
|  |  | **(0.016, 0.045)** | (-0.024, 0.048) | (-0.021, 0.006) | (-0.022, 0.006) |
| **Month** | *February* | **0.054*** |  |  |  |
| *(Reference = January)* |  | **(0.006, 0.101)** |  |  |  |
|  | *March* | **0.262***** |  |  |  |
|  |  | **(0.199, 0.324)** |  |  |  |
|  | *April* | **0.168***** |  |  |  |
|  |  | **(0.106, 0.229)** |  |  |  |
|  | *May* | **0.253***** |  |  |  |
|  |  | **(0.198, 0.307)** |  |  |  |
|  | *June* | **0.266***** |  |  |  |
|  |  | **(0.209, 0.324)** |  |  |  |
|  | *July* | **0.249***** |  |  |  |
|  |  | **(0.188, 0.311)** |  |  |  |
|  | *August* | **0.272***** |  |  |  |
|  |  | **(0.219, 0.325)** |  |  |  |
|  | *September* | **0.306***** |  |  |  |
|  |  | **(0.243, 0.368)** |  |  |  |
|  | *October* | **0.236***** |  |  |  |
|  |  | **(0.187, 0.285)** |  |  |  |
|  | *November* | **0.267***** |  |  |  |
|  |  | **(0.213, 0.321)** |  |  |  |
|  | *December* | **0.073**** |  |  |  |
|  |  | **(0.023, 0.123)** |  |  |  |
| **Constant** |  | **2.536***** | **2.912***** | **0.871***** | **0.871***** |
|  |  | **(2.308, 2.764)** | **(2.496, 3.327)** | **(0.830, 0.911)** | **(0.829, 0.912)** |
| N | *Observations* | 49,171 | 7,858 | 5,529 | 5,529 |
|  | *Facilities* | 989 | 482 | 479 | 479 |
|  | *Transition* | 585 | 128 | 126 | 126 |
|  | *Maintenance* | 404 | 354 | 353 | 353 |

*p<0.05, **p<0.01, ***p<0.001
